# Erlotinib Improves the Response of Glioblastoma Cells Resistant to Photodynamic Therapy

**DOI:** 10.3390/brainsci14121192

**Published:** 2024-11-26

**Authors:** Karen Olthoff, Ayelén D. Nigra, Laura N. Milla Sanabria

**Affiliations:** Departamento de Biología Molecular, Facultad de Ciencias Exactas, Físico-Químicas y Naturales, Universidad Nacional de Río Cuarto (UNRC), INBIAS (CONICET-UNRC), Río Cuarto 5800, Argentinaanigra@exa.unrc.edu.ar (A.D.N.)

**Keywords:** glioblastoma, photodynamic therapy, resistance, EGFR, erlotinib

## Abstract

**Background:** Glioblastoma (GBM) is the most common and deadly type of brain cancer in adults. Dysregulation of receptor tyrosine kinase pathways, such as the epidermal growth factor receptor (EGFR), contributes to therapeutic resistance. Drugs that inhibit tyrosine kinase activity and monoclonal antibodies against EGFR are strategies used in clinical trials. Photodynamic therapy (PDT) is a tumor treatment that involves the administration of a photosensitizing drug, followed by its activation with visible light, which causes cell death due to oxidative stress. Although PDT helps prolong median survival in patients with GBM, complete remission has not been achieved. Populations of GBM cells have been obtained from the T98G line resistant to PDT with methyl-5-aminolevulinic acid (Me-ALA) for characterization, comparing them with the original parental population. **Objective:** The objective of this work was to evaluate the general response of T98G GBM cells resistant to PDT when EGFR activity is inhibited with the drug erlotinib. **Methods and Results:** It has been observed that the administration of the EGFR inhibitor drug in combination with PDT reduced viability (MTT) in resistant populations compared to PDT alone. Furthermore, the PpIX content (flow cytometry) was increased in the resistant population when cells were incubated with Me-ALA and erlotinib. Erlotinib prevented cell proliferation of parental and resistant spheroids. Wound closure was reduced in both parental and PDT-resistant populations. **Conclusions:** Our results indicate that EGFR activation would be relevant in the resistance of GBM cells to PDT.

## 1. Introduction

Glioblastoma (GBM) is a high-grade glioma (grade IV). This represents one of the most malignant types of cancer and the most common malignant primary tumor of the central nervous system. GBM patients have a median survival of less than 15 months from initial diagnosis [1]. Standard therapies for GBM are based on surgical removal followed by radiotherapy and chemotherapy with the drug temozolomide (TMZ). Despite maximal surgical resection complemented by the adjuvant therapies, almost all GBM tumors locally recur after treatment. Current challenges for the treatment of GBM include its incomplete resection, a high degree of genetic heterogeneity, the blood–brain barrier, and an immunosuppressive tumor microenvironment. Surgical resection of GBM is almost impossible due to its highly invasive nature. Although tumor margins can be roughly determined by imaging, GBM grows with microscopic finger-like projections, imperceptible to presurgical or even intraoperative imaging methods [2].

Fluorescence-guided resection (FGR) allows for better visualization of tumor tissue and prolongs patient survival. In the last two decades, several fluorescent compounds have been studied for FGR, such as 5-aminolevulinic acid (5-ALA) [3]. 5-ALA is a prodrug which is taken up by cells and metabolized in the heme biosynthesis pathway to protoporphyrin IX (PpIX), an endogenous photosensitizer. Finally, the enzyme ferrochelatase incorporates iron into PpIX to form heme. PpIX accumulates in cancer cells after 5-ALA administration due to abnormalities in the heme pathway, such as the poor ferrochelatase activity [4]. Due to the fluorescence property of the photosensitizer PpIX, 5-ALA is used in the clinic for photodiagnosis and FGR for gliomas [3]. Its intraoperative advantages have been shown to increase the surgical resection area and to prolong overall survival [5].

Following the US Food and Drug Administration’s (FDA) approval of ALA for FGR of high-grade gliomas, there has been renewed interest in using this PpIX precursor for photodynamic therapy (PDT) in these tumors. PDT is a treatment that uses photosensitizers and light to generate reactive oxygen species (ROS), such as singlet oxygen (^1^O_2_), superoxide radicals (·O2−) and hydroxyl radicals (·OH). The ROS generated cause the death of tumor cells, immune response, and microvascular damage. The use of PDT with Photofrin^®^ (porfimer sodium) increased in several countries (United States, Canada, Australia, the United Kingdom and Austria), as a result of the positive therapeutic results of the first trial in patients with glioma carried out by Perria et al. in 1980 in Italy [6]. PDT was approved in Japan in 2013 as an intraoperative treatment for malignant gliomas. Different clinical applications of PDT in patients with GBM used Photofrin^®^, Foscan^®^ (m-Temoporfin), Laserphyrin^®^ (talaporfin sodium), and Gliolan^®^ (5-ALA) [7].

The first molecular alterations in GBM were found in isocitrate dehydrogenase IDH1/2, which were used for categorization and prognostication of these tumors. Other alterations in primary GBMs were recognized in the proto-oncogenes EGFR, PDGFR, MET, CDK4, CDK6, CCND1, CCDN3, MDM2, MDM4, and MYCC and the tumor suppressor genes TP53, RB1, CDKN2A (p16INKA4/p14ARF), and PTEN [8]. Epidermal growth factor receptor (EGFR) is a transmembrane tyrosine kinase encoded by a gene located on chromosome band 7p12. EGFR is a membrane of the ErbB receptors family. The amplification and over-expression of this receptor were associated with tumor cell proliferation, angiogenesis, tumor invasion, and therapeutic resistance to chemotherapy and radiotherapy [9]. EGFR has a kinase domain composed of the N and C lobes and an adenosine triphosphate (ATP) binding cleft between the two lobes. When a ligand binds to its EGFR receptor, an asymmetric dimer is formed and the phosphate of ATP is transferred to the tyrosine residue of the regulatory domain. Several proteins are bound to this phosphorylated tyrosine and signals are transmitted downstream by the rat sarcoma (RAS)-rapidly accelerated fibrosarcoma (RAF)-mitogen-activated protein kinase (MAPK) and phosphatidylinositol 3-kinase (PI3K)-protein kinase B (PKB/AKT) pathways. EGFR tyrosine kinase inhibitors (TKIs) competitively inhibit ATP with cleft of the kinase domain [10]. For GBM, clinical trials have been developed inhibiting the EGFR using drugs, peptide vaccines, and antibodies [11].

Despite growing knowledge about the causes of failure of oncological treatments against GBM, there is still an enormous need for therapeutic advances that open hope for improving the quality of life and prolonging the survival of patients [12]. Deepening GBM resistance studies are promising for a better understanding of the causes of relapses and offer a basis for the development of more effective therapeutics [13]. Populations of GBM cells were obtained from the T98G line resistant to PDT with a methylated derivative of ALA (Me-ALA) for characterization compared with the original parental population. We had previously determined by RT-qPCR that the resistant cells have increased EGFR mRNA levels compared to the parental populations [14]. The objective of this work was to evaluate the general response of T98G GBM cells resistant to PDT when EGFR activity is inhibited with the drug erlotinib.

## 2. Materials and Methods

### 2.1. Cell Culture

T98G (ATCC) cells were cultured in high glucose Dulbecco’s Modified Eagle’s medium (DMEM; Gibco, Life Technologies Corporation, NY, USA), supplemented with 10% *v/v* fetal bovine serum (Internegocios S.A., Buenos Aires, Argentina) and 1% *v/v* antibiotic-antimicotic (penicillin 10,000 units/mL, streptomycin 10,000 μg/mL, amphotericin B 25 μg/mL; Gibco, Life Technologies Corporation, NY, USA). Cell cultures were incubated at 37 °C in an atmosphere containing 5% CO_2_.

### 2.2. Photosensitizer

The pro-drug used in this work was the methyl-5-aminolevulinic acid (Me-ALA; Sigma-Aldrich, Merck S.A., Buenos Aires, Argentina) as the precursor of the photosensitizer PpIX. A stock solution of 100 mM Me-ALA was made in sterile phosphate buffered saline (PBS), from which 1 mM work solution was made employing culture medium (DMEM) without serum.

### 2.3. Light Source

For PDT treatment, cells were irradiated employing a monochromatic light source (635 nm ± 17 nm) with a multi-LED system (coherent light) at an irradiation intensity of 16.9 mW/cm^2^ (as measured by Coherent Lasermate power meter).

### 2.4. Obtainment of PDT-Me-ALA Resistant Cells

T98G resistant to PDT/Me-ALA cells were obtained in a similar way to that described in Vilchez et al., 2021 [14]. Resistant populations to eight rounds of PDT have been obtained. In the procedure, an irradiation dose that allows only a low percentage of cells to survive was used (lethal dose 70–80 = 8.60 J/cm^2^). T98G cells were cultured in 35 mm plates (*n* = 5) and incubated with Me-ALA 1 mM for 4 h. Thereafter, cells were exposed to irradiation with red light. The surviving cells were harvested and replated at 24–48 h after PDT. Once they proliferated, cells were submitted to a new PDT treatment. As the number of rounds of PDT increased, less cell death was observed. The final population received a total of 8 rounds of PDT, at which time no death was observed. The initial population (never received PDT) was called the parental population; the cellular population submitted to one PDT treatment was called the first resistant generation; the following generations were named consecutively. Once the 8th resistant generation was obtained, cells were kept in frozen stocks. To check the resistance ability of the 8th resistant generation with respect to the parental population, the cells were defrosted and a cell viability assay (3-[4,5-dimethylthiazol-2-yl]2,5-diphenyltetrazolium bromide; MTT) was made after PDT treatment with several irradiation doses as indicated in the next section.

### 2.5. Viability Assay by MTT

#### 2.5.1. Resistance Determination

The grade of resistance to PDT was determined in T98G 8th generation cells (“Resistant cells”), with respect to parental cells. 12 × 10^4^ cells/mL of each population were seeded in multiwell-96 plates (P96). After 24 h, cells were incubated with Me-ALA 1 mM for 4 h at 37 °C and then irradiated at light doses of 5.07, 6.08, and 8.11 J/cm^2^ (*n* = 8). Cells with drug but without light were employed as drug controls and cells with light (at 8.60 J/cm^2^) but without drug were employed as light controls. Cells without drug and without light were employed as controls. After treatments, culture medium was replaced by complete medium. After 24 h of PDT, the cell viability was determined by MTT assay. 10 μL of MTT solution (Sigma-Aldrich; 5 mg/mL PBS) was added to the cells in each well and it was incubated for 3–4 h. The culture medium was removed, and formazan crystals were suspended in DMSO (Cicarelli ^®^, Santa Fé, Argentina). Measures of the absorbance were taken at 540 nm with a spectrophotometer (Multiskan FC, Thermo Fisher Scientific, Shanghai, China). The average absorbance values of the controls were expressed as 100% viability. The average absorbance values of all groups were relative to the controls. Results are reported as the mean ± standard error of mean (SEM). The experiment was performed 3 times. Results of a representative experiment are shown.

#### 2.5.2. Cell Viability After Combined Treatment of PDT and Erlotinib

Parental and resistant T98G cells were seeded on P96 plates (12 × 10^4^ cells/mL). The next day the cells were incubated with erlotinib at doses of 9.20, 25 and 50 µM (*n* = 8) [15]. Cells without drugs were used as controls. DMSO vehicle control groups were included. At 24 h the cell viability was analyzed by MTT assay, as described in the previous section. Also, cell viability was determined after combined treatment of PDT with erlotinib. The erlotinib dose of 50 µM was selected based on previous works that have evaluated its effect on cancer cell lines, such as GBM [15], pancreatic adenocarcinoma [16], and lung cancer [17], as well as in human CD34 cells-phlebotomized units of blood for the study of the treatment of Polycythemia Vera (myeloproliferative neoplasm) [18].

Following Bagherian et al., who administered 50 μM curcumin and 50 μM erlotinib together in U87 GBM cells for in vitro co-delivery [15], co-administration of Me-ALA and erlotinib was performed in T98G parental and resistant populations. Cells were incubated with Me-ALA (1 mM) and erlotinib (50 µM) for 4 h and then irradiated at 6.08 and 8.11 J/cm^2^. After irradiation, the medium was replaced by a complete medium. Erlotinib was added in erlotinib and erlotinib + PDT groups. After 24 h, cell viability was measured by MTT. Results are reported as the mean ± standard error of mean (SEM). The experiment was performed 3 times. Results of a representative experiment are shown.

### 2.6. Flow Cytometry

The fluorescence of the photosensitizer PpIX was measured by flow cytometry in parental and resistant T98G populations after incubation with Me-ALA and erlotinib. Cells (20 × 10^4^ cells/mL) were seeded in Petri dishes (35 mm plates) (*n* = 6). After 24 h, cells were incubated with Me-ALA (1 mM) and erlotinib (50 µM) for 4 h, washed with PBS three times, trypsinized, resuspended in complete medium (to stop the effect of trypsin) and centrifuged. The pellet was resuspended in PBS and the cellular PpIX fluorescence was measured by flow cytometry (Guava^®^ easyCyte System; EMD Millipore Corp., Billerica, MA, USA). Excitation wavelength of 488 nm (blue laser) was used and the emission at 670 nm was determined (Red B). Flow cytometry data were analyzed employing FlowJo 7.6 and PpIX fluorescence histograms were compared. The percentage of positive cells for the photosensitizer fluorescence and the media fluorescence intensity were determined. Results are reported as the mean ± SEM. The experiment was performed 3 times. Results of a representative experiment are shown.

### 2.7. 3D-Cultures Proliferation

T98G parental and resistant cells were seeded in P96 U-bottom wells coated with agarose (1% in deionized H_2_O) [19]. A total of 2000 cells/well were seeded in DMEM medium 10% serum with erlotinib (50 µM; *n* = 12). Controls without drug and vehicle controls with DMSO were included. The spheroids were photographed (Nikon DS-Qi1MC; Nikon Corp., Tokyo, Japan) 3 days after being seeded, and the number of cells by spheroids was counted by employing a Neubauer chamber. For cell counting, a 0.4% trypan blue solution was used. Results are reported as the mean ± SEM. The experiments were performed 3 times. Results of a representative experiment are shown.

### 2.8. Wound Healing Assay 

Parental and resistant T98G cells were seeded (24 × 10^4^ cells/mL) in P24. The next day, with a confluence of 100%, wounds were made in the cell monolayer using a micropipette tip (2 wounds per well). The cells were rinsed 3 times with a complete medium to remove lifted cells. Then, the cells were cultured with erlotinib at doses of 25 and 50 µM using culture medium with 1 and 10% serum (*n* = 6 wells). Cells without erlotinib and without DMSO were used as controls. Vehicle control groups with DMSO were included. When the control group closed the wounds, the cells were fixed with cold methanol and stained with toluidine blue (0.05%) for better image visualization. Photographs were taken (Nikon DS-Qi1MC, Nikon Corp., Japan) at day 0 (t0) and final time (tf). The wound area was measured using ImageJ 1.53t (*n* = 8 wound areas). The percentage of wound closure was calculated using the formula: % wound closure = [100 − (wound area tf/wound area t0)] × 100. Results are reported as the mean ± SEM. The experiments were performed 3 times. Results of a representative experiment are shown.

### 2.9. Statistical Analysis

The values in the figures are expressed as mean ± SEM. The R and R Studio software (R4.3.1) was used to carry out the statistical analyses. The statistical significance of the differences between the means was determined with the analysis of variance (ANOVA). When the means were found to be significantly different (with a *p*-value less than 0.05) multiple pairwise comparisons were performed using Tukey’s HSD test.

## 3. Results

### 3.1. T98G Cells Exposed to Eight Cycles of PDT/Me-ALA Had a High Degree of Resistance to Treatment

**Figure 1:**
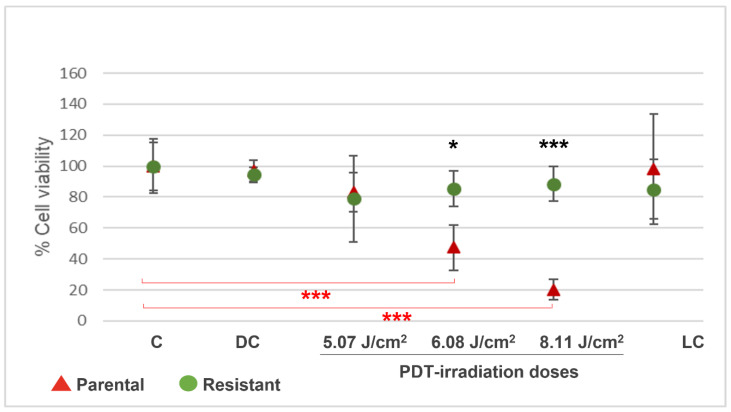
Percentages of cell viability by MTT after PDT in T98G parental and PDT-resistant populations. Parental and resistant T98G cells were incubated with Me-ALA for 4 h and irradiated at 5.07, 6.08, and 8.11 J/cm^2^. Resistant populations had high viability at irradiation doses lethal to the parental populations. C = control (without Me-ALA, without light); DC = drug control (with Me-ALA, without light); LC = 8.11 J/cm^2^ light control (without Me-ALA, with light, 8.11 J/cm^2^); photodynamic therapy (PDT)-treated (Me-ALA + 5.07 J/cm^2^; Me-ALA + 6.08 J/cm^2^; Me-ALA + 8.11 J/cm^2^). * *p* < 0.05; *** *p* = 0.001 (resistant respect to parental cells, black asterisks), *** *p* ≤ 0.001 (resistant with PDT respect to resistant C, red asterisks). Results are reported as the mean ± standard error of mean (SEM). The experiment was performed 3 times. Results of a representative experiment are shown.

In order to obtain the PDT-resistant GBM populations to perform the experiments, T98G cells were exposed to eight rounds of PDT with Me-ALA (similar to our publication Vilchez et al., 2021 [14]). Cell viability around 80–90% was observed in resistant cells at 5.07, 6.08, and 8.11 J/cm^2^ irradiation doses (Figure 1), while the parental populations present viability of around 80, 50, and 20% at these doses, respectively.

### 3.2. PDT in Combination with Erlotinib Reduced Viability in Resistant Populations Compared to PDT Alone

**Figure 2:**
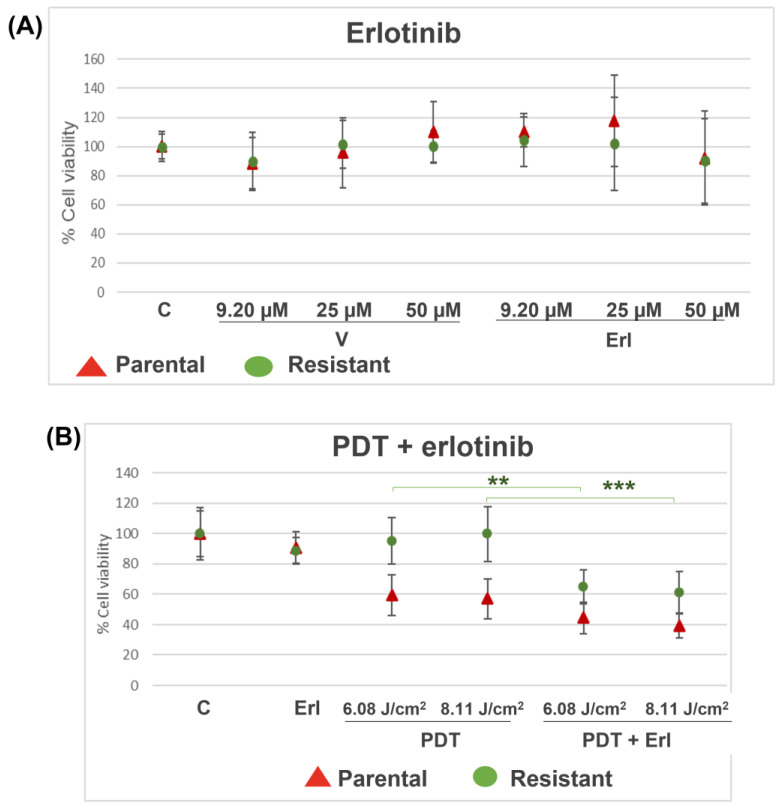
(**A**) Percentages of cell viability by MTT in parental and resistant T98G cells incubated with erlotinib. Parental and resistant T98G cells were incubated with erlotinib (Erl) for 24 h at doses of 9.2, 25, and 50 µM. Erlotinib and its vehicle DMSO (V) did not affect the cell viability (at 24 h) of parental and resistant T98G populations. (**B**) Cell viability by MTT after PDT and erlotinib combination in parental and resistant T98G cells. Cells were incubated with Me-ALA (1 mM) and erlotinib (50 uM) for 4 h and then irradiated at 6.08 and 8.11 J/cm^2^. After 24 h, cell viability was measured by MTT. PDT in combination with erlotinib reduced viability in resistant populations compared to PDT alone. Photodynamic therapy (PDT)-treated (Me-ALA + 6.08 J/cm^2^; Me-ALA + 8.11 J/cm^2^). ** *p* < 0.01; *** *p* = 0.001 (resistant populations, green asterisks). Results are reported as the mean ± standard error of mean (SEM). The experiment was performed 3 times. Results of a representative experiment are shown.

The EGFR inhibitor drug erlotinib and its vehicle (DMSO) did not affect the cell viability of parental and resistant T98G populations at the analyzed doses of 9.20, 25, and 50 µM at 24 h (Figure 2A). A dose of 50 µM was used to analyze cell viability when PDT was combined with erlotinib. The resistant population viability was reduced from values of around 100% with PDT (1 mM Me-ALA; 6.08 and 8.11 J/cm^2^) to around 60% when PDT was combined with erlotinib. In the parental population, a slight reduction in viability was observed when the cells were incubated with the EGFR inhibitor drug, but it was not statistically significant (Figure 2B).

### 3.3. Erlotinib Increases the PpIX Content from Me-ALA in Resistant Populations

**Figure 3:**
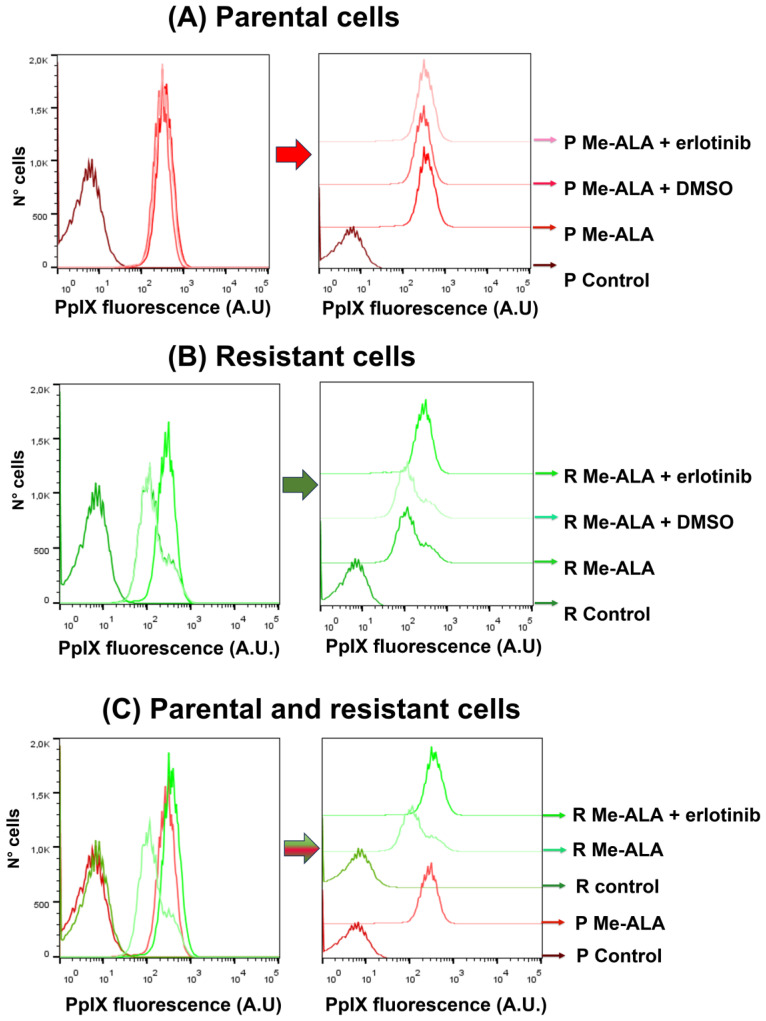

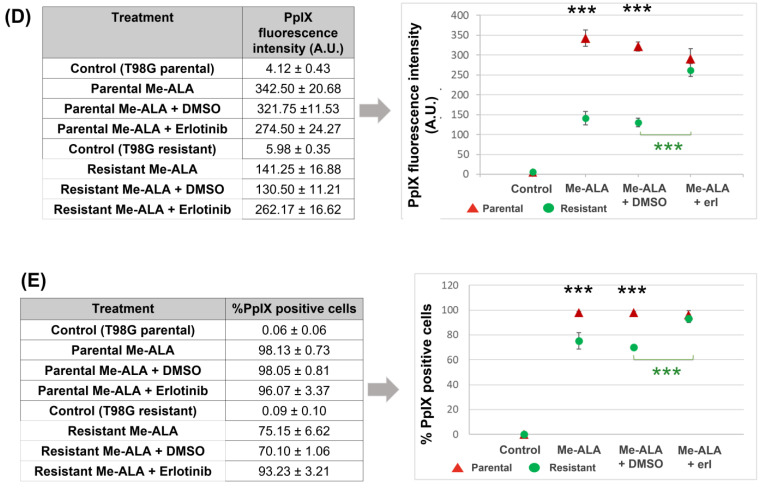
Flow cytometry of PpIX content after Me-ALA and erlotinib incubation. Parental (**A**) and resistant cells (**B**) were incubated with Me-ALA (1 mM) and erlotinib (50 µM) for 4 h. PpIX fluorescence was measured by flow cytometry. Erlotinib increased the content of PpIX in the resistant population (**B**–**E**). C = control (without Me-ALA, without erlotinib); Erl = erlotinib; A.U. = arbitrary units. *** *p* < 0.001 (resistant respect to parental cells, black asterisks), *** *p* < 0.001 (resistant cells, green asterisks). Results are reported as the mean ± SEM. The experiment was performed 3 times. Results of a representative experiment are shown.

In order to know if erlotinib has an effect on the PpIX content in parental and resistant cells when incubated with Me-ALA, the cells were seeded in 35 mm dishes. The next day they were incubated with erlotinib and Me-ALA for 4 h. The content of the photosensitizer PpIX was determined by flow cytometry. In the groups with Me-ALA and without erlotinib, the amount of photosensitizer was lower in the resistant populations compared to the parental cells, as it was previously determined in our publication [10]. Erlotinib increased the content of PpIX from Me-ALA in the resistant population, but not in the parental cells (Figure 3A,B). With the incubation of the EGFR inhibitor drug, the content of the photosensitizer PpIX in resistant populations was equal to the PpIX content found in parental cells (Figure 3C–E).

### 3.4. Erlotinib Inhibits Proliferation of 3D Cultures in Parental and Resistant T98G Populations

**Figure 4:**
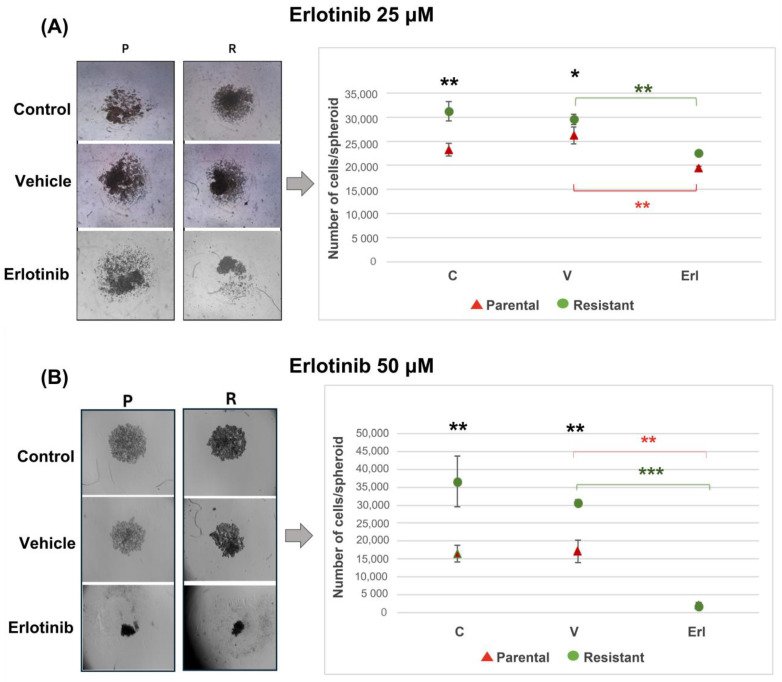
Parental and resistant T98G spheroids incubated with erlotinib. Photographs and cell number count per spheroid 3 days after seeding 2000 cells per well in U-bottom plates with agarose. Cells were seeded and incubated with erlotinib (Erl) at the dose of 25 µM (**A**) and 50 µM (**B**) for 3 days. Erlotinib inhibited the proliferation of parental and resistant T98G 3D cultures. C = control, V = vehicle, Erl = erlotinib. * *p* < 0.01; ** *p* < 0.01 (resistant respect to parental cells, black asterisks); ** *p* < 0.01 (parental cells, green asterisks; resistant cells, red asterisks) and *** *p* = 0.001 (parental cells, green asterisks). Results are reported as the mean ± SEM. The experiment was performed 3 times. Results of a representative experiment are shown.

In order to know the proliferation capacity of T98G parental and resistant cells when the EGFR is inhibited, 3D cultures were performed (2000 cells/spheroid) and the number of cells per spheroid was counted 3 days after plating. The resistant spheroids had twice the proliferation compared to the parental spheroids (16,458 ± 4066 cells/spheroid in parental population; 36,612 ± 7141 cells/spheroid in resistant population). Parental and resistant spheroids did not proliferate when they were incubated with erlotinib 50 µM (2187 ± 1362 cells/spheroid in parental population; 1770 ± 650 cells/spheroid in resistant population) (Figure 4B). The proliferation of spheroids from both populations was reduced when incubated with erlotinib at 25 μM, with less effect than with the highest dose of the drug (Figure 4A). At the erlotinib dose of 50 µM, the DMSO vehicle showed some inhibition effect on proliferation, although this was not statistically significant (Figure 4B). At the erlotinib dose of 25 µM, no effect of the vehicle was observed (Figure 4A).

### 3.5. Erlotinib Reduced Wound Closure in Parental and Resistant T98G

**Figure 5:**
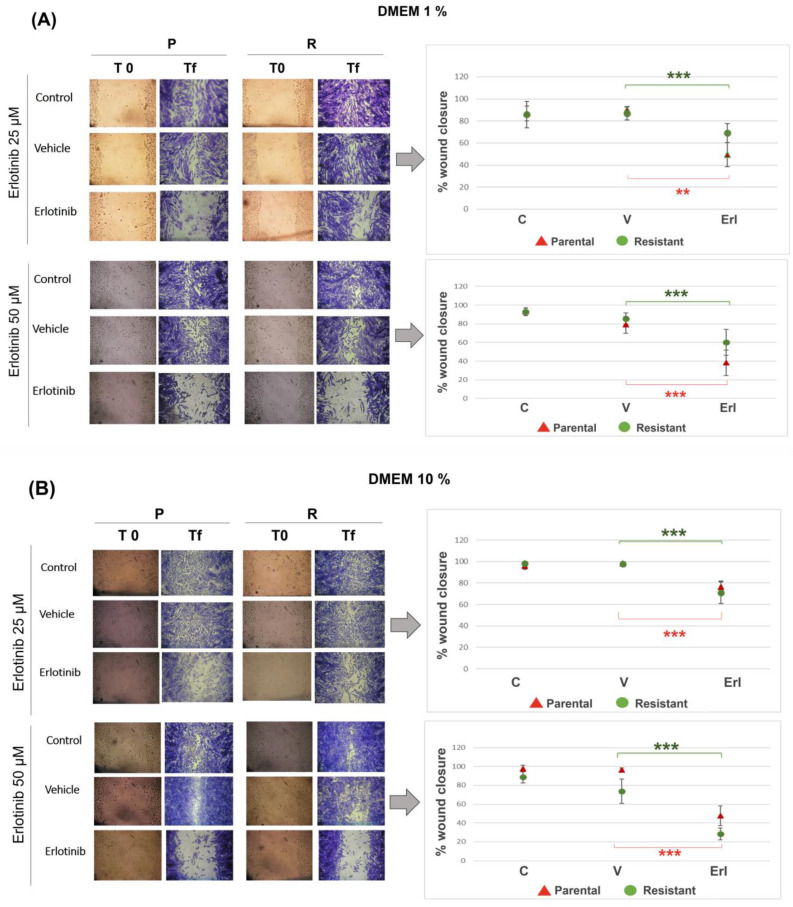
Wound closure in parental and resistant T98G populations incubated with erlotinib. Immediately after opening the wounds, cells were incubated with erlotinib (Erl) at the dose of 25 µM in DMEM 1% (**A**) and DMEM 10% (**B**) and at the dose of 50 µM in DMEM 1% (**A**) and DMEM 10% (**B**). Photographs of the wounds were taken at day 0 and at the final time. A quantification of the percentage of wound closure was performed using ImageJ 1.53t. Erlotinib reduced wound closure similarly in parental and resistant T98G, both when 1% DMEM was used and when 10% DMEM was used. C = control, V = vehicle, Erl = erlotinib. ** *p* < 0.01, *** *p* < 0.001 (parental cells, green asterisks; resistant cells, red asterisks). Results are reported as the mean ± SEM. The experiment was performed 3 times. Photographs and results of a representative experiment are shown.

To determine the effect of erlotinib on wound closure capacity, wounds were made on confluent monolayers of parental and resistant T98G populations. DMEM medium with 1% and 10% serum was used (employing low percentages of serum cell proliferation is minimized, while with high percentages of serum the cells have a high proliferation rate). Erlotinib reduced wound closure similarly in parental and resistant T98G, both when 1% DMEM was used (Figure 5A) and when 10% DMEM was used (Figure 5B). The percentage of wound closure was lower for the 50 µM dose compared to the 25 µM dose. At the 50 µM dose, a slight effect on wound closure was observed due to the DMSO vehicle, although this was not statistically significant (except for 1% DMEM-parental cells).

Using 1% DMEM, when the wound closure percentage reached 90% in the control group (at 4 days), the parental populations treated with 25 µM erlotinib had 70% wound closure and the resistant cells had 50% (without statistically significant differences between parental and resistant cells). Parental populations treated with 50 µM erlotinib had 60% wound closure and resistant cells 40% (no statistically significant differences) (Figure 5A). Using 10% DMEM, when the wound closure percentage reached 90–100% in the control group (at 2 days), the parental and resistant populations treated with 25 µM erlotinib had around 70%. Parental populations treated with 50 µM erlotinib had 30% wound closure and resistant cells 50% (Figure 5B).

## 4. Discussion

GBM is a grade IV brain tumor which represents one of the most lethal human cancers. Standard therapies for GBM consist of surgical resection followed by chemotherapy and radiotherapy [2]. A meta-analysis reported by Ren et al., including 1294 patients from 31 articles, shows that the fluorescence guided resection (FGR) and PDT employing ALA allow a significant increase in the maximum resection rate and prolong the patient survival after PDT [20].

Advancement in precision oncology has renewed attention to the study of the receptor tyrosine kinase (RTK) EGFR as an important therapeutic target for GBM [21]. Amplifications in the EGFR gene are detected in 57.4% of primary GBM patients, leading to high levels of EGFR protein and it has been observed that amplifications of the EGFR are retained in recurrent gliomas [22]. Repeated cycles of oncological treatments in vitro can be performed with the aim of amplifying the biochemical changes associated with cell resistance and to identify a selective target on surviving cells [23]. We have found that PDT-resistant GBM cells from the T98G line have high levels of mRNA of EGFRs, form spheroids with a greater number of cells in vitro, and are more tumorigenic when injected into mice, compared to the parental population [14].

Based on the characteristic of high levels of EGFR mRNA of the resistant T98G populations in relation to the parental cells, we set the objective of evaluating the general response of T98G GBM cells resistant to PDT when EGFR activity is inhibited with the drug erlotinib. The relatively high viability to PDT of resistant populations and high proliferation of resistant spheroids [14] could be related to the expression of EGFR. On the one hand, we analyzed whether erlotinib affects the viability of resistant cells when PDT is administered. On the other hand, we determined whether erlotinib alters the proliferation of 3D cultures and the wound closure capacity (2D assay).

PDT in combination with erlotinib reduced viability in resistant populations compared to PDT alone, reaching viability levels similar to the parental cells. These results were related to an increase in the content of the photosensitizer PpIX in the resistant population when the cells were incubated with Me-ALA in combination with erlotinib. Fontana et al. found that EGFR inhibition increases the PpIX in GBM cells incubated with ALA because the expression of the enzyme heme oxygenase-1 (HO-1) is reduced. They showed that increased EGFR activity results in upregulation of HO-1-mediated heme clearance and ultimately reduced PpIX fluorescence. HO-1 accelerated heme depletion and thus a shift in enzymatic activity occurs in favor of increased PpIX metabolism by ferrochelatase. It is known that the expression of the HO-1 is primarily regulated at the transcriptional level by activating transcription factors such as NF-kβ, AP-2 and the heat shock-responsive element (HSE). EGF induces NF-kB activation through multiple EGFR dependent signaling molecules, including PI3K, protein kinase C (PKC), and IKK signaling pathways. So, EGF/EGFR signaling promotes HO-1 expression and activity in GBM cells through activation of the PI3K/AKT/NF-κB cascade [24]. We could hypothesize that this axis, which leads to a lower accumulation of the photosensitizer PpIX, has been favored in the selective obtaining of resistant populations, among other resistance mechanisms. Then, the inhibition of EGFR activity by erlotinib leads to the increase in PpIX in resistant populations and to the consequent decrease in cell viability after PDT.

As we can see in the T98G 3D cultures, erlotinib inhibited/reduced the proliferation of both the parental T98G populations and the resistant populations. It could initially be expected that resistant cells would have a lower response to erlotinib in relation to their proliferation in 3D cultures. However, resistant cell spheroids were equally sensitive to the drug in relation to the parental cells with the doses of erlotinib used. With the 25 µM dose of the drug, spheroids had lower number of live cells. The same number of living cells was found with erlotinib treatment at the dose of 50 µM as at the beginning of seeding the spheroids. The doses of erlotinib used would be effective in inhibiting EGFR in resistant populations, reducing or stopping the proliferation of spheroids.

Also, erlotinib reduced wound closure in parental and resistant T98G using culture medium with low and high content of fetal bovine serum, suggesting that the decrease in EGFR activity could generate an effect on the cells leading to reduced migration, proliferation, or even death. It is known that the EGFR is activated by ligands (EGF, EREG, and EPGN) which induce dimerization structures and activate downstream effector pathways, including MAPK, PI3K, and STAT3, by auto-phosphorylation of tyrosine residues in its cytoplasmic domain [21]. In GBM, the activation of these pathways leads to tumor growth, invasion, angiogenesis, and drug resistance to radio- and chemotherapy [22]. A large body of work shows that EGFR inhibition reduces GBM cell migration, invasion, and proliferation. Ji et al. found that miR-615 expression was downregulated in GBM cells and tissues. They found that miR-615 plays a tumor suppressor role in GBM cell proliferation, migration, and invasion by targeting EGFR expression. miR-615 could be a novel biomarker for early diagnosis or therapeutic targets of GBM [25]. Liu et al. analyzed the anti-tumor activity and effectiveness of the third-generation EGFR-targeted drug AZD9291 in vitro and in an orthotopic GBM model. AZD9291 significantly inhibited colony formation, migration, and invasion of GBM cells and reduced tumor growth and prolonged mice survival [26]. Based on our initial results, it can be thought that EGFR inhibition could be necessary during PDT to eliminate resistant cells that overexpress that receptor and after treatment to inhibit/decrease the proliferation of resistant cells that can escape PDT.

Similar to T98G cells, in our work on PDT-resistant squamous cell carcinoma populations, we found that PDT-resistant cells of the SCC-13 line accumulated less PpIX and were more tumorigenic than the parental cells [27]. Furthermore, we determined that resistant SCC-13 populations have amplifications in the gene that encodes the mitogen-activated kinase MAPK/ERK, while these amplifications are not found in the parental SCC-13 population. These DNA changes were also observed at the mRNA and protein expression level and correlated with an increase in the expression and activation of the EGFR. These results agree with those obtained by immunohistochemistry of human biopsies of squamous cell carcinomas resistant to two sessions of PDT with Me-ALA. Thus, our studies in squamous carcinoma propose that genomic imbalances related to the EGFR and MAP3K1 may be an important factor in resistance to PDT [28].

In this work, our trials were carried out using erlotinib with the main objective of inhibiting EGFR activity to analyze the response of resistant GBM cells. Our project aims to use a plasmid that directly inhibits the expression of EGFRs at the protein level to perform PDT studies. Finally, we will move to an in vivo GBM model. We will use different GBM cell lines, analyze the presence and frequency of mutations in the EGFR, as well as molecular mechanisms involved in the response to combined treatment (PDT-EGFR inhibition). The presence of various mutations in the EGFR receptor hinders the effectiveness of therapies in the clinic and constitutes a key problem in oncology against GBM [10]. For this reason, several novel agents, such as other small molecule inhibitors and antibodies, are being studied as a strategy to eliminate each EGFR variant that may be found in patients’ tumors [22,29]. The aim is to select personalized treatments based on the molecular characteristics of the tumor. Hope is found in precision oncology to target the EGFR of GBM (among other markers) to perform personalized treatments, improve blood–brain barrier crossing, and explore combination therapies [30]. Studies on the EGFR in GBM are expected to lead to improved outcomes in this highly vulnerable patient population [10].

The development of new delivery methods and nano-drugs aims to ensure more selective drug delivery to the brain. Currently, there is strong research on the development of different strategies to direct compounds more selectively towards cancer cells, reducing their toxicity in normal tissue. To decrease the toxicity and improve the effectiveness of oncological drugs, whose use at high doses is limited because of its adverse effects, carriers could be used to deliver drugs to neoplastic tissues. Drug-containing nanoparticles can reduce side effects caused by drugs by encapsulation and release in the tumor [31].

## 5. Conclusions

**Figure 6:**
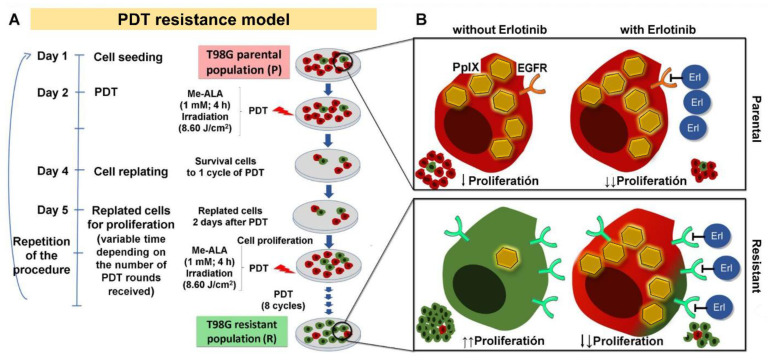
Protocol for obtaining PDT-resistant T98G cells and the effect of erlotinib. (**A**) Timeline: initial population of T98G cells (parental) is treated with PDT (Me-ALA); the surviving cells are amplified forming a population of resistant cells to one cycle of PDT. The process is repeated for 8 cycles (a selected population resistant to PDT is formed). (**B**) PDT-resistant T98G cells overexpress EGFR mRNA, accumulate less PpIX and have increased 3D proliferation with respect to parental cells [14]. Treatment with the EGFR inhibitor erlotinib (erl) causes a decrease in 3D cell proliferation (parental and resistant populations). Additionally, when treated with erlotinib, resistant cells regain the ability to accumulate PpIX becoming sensitive to PDT.

In this work we have observed that the inhibition of EGFR activity using the small molecule erlotinib reduced the viability of GBM populations of the T98G line resistant to PDT with Me-ALA. This could be explained, at least in part, by the increase in the PpIX content in those resistant populations when EGFR is inhibited (Figure 6). When erlotinib is used in resistant populations, they behave similarly to the parental cells without erlotinib in terms of viability in response to PDT and PpIX content. Also, erlotinib reduced the proliferation of 3D cultures and reduced the wound closure, in both parental and resistant cells. In GBM, inhibition of EGFRs could be important, not only to reduce proliferation, but also to improve PDT results by increasing the amount of the photosensitizer in groups of cells that accumulate low less PpIX. Figure 6 summarizes the model for obtaining PDT-resistant populations used in this study and the effect of erlotinib on 3D proliferation of parental and resistant cells.

## Data Availability

The data presented in this study are available on request from the corresponding author due to the need for a formal data sharing agreement.
